# Ghost in the right atrium: A case report on successful identification of residual fibrous tissue

**DOI:** 10.1016/j.heliyon.2023.e21982

**Published:** 2023-11-03

**Authors:** Yaru Li, Luyang Jiang, Lu Wang, Qiaoyu Han, Xinrui Yin, Yi Feng

**Affiliations:** Department of Anesthesiology, Peking University People's Hospital, Beijing, China

**Keywords:** Ghost, Transesophageal echocardiography, Artificial cardiac pacing, Transvenous lead extraction (TLE)

## Abstract

The term "ghost" refers to residual fibrous tissue that remains within the cardiovascular system following the removal of implanted device leads. In this case, a 75-year-old male underwent a transvenous lead extraction procedure prompted by an infection. After the procedure, transesophageal echocardiography (TEE) revealed a stable "ghost" in the right atrium. Figures and videos dynamically depicted the ghost's morphology, clearly showing that one end of this spectral entity was firmly attached to the atrial wall, while the other end displayed unrestrained mobility within the bloodstream. After undergoing a thorough evaluation that confirmed the benign nature of the "ghost", there was evidently no need for medical intervention. In summary, the article portrayed a rare yet intriguing "ghost" observed during the perioperative period. Furthermore, this study emphasizes the crucial significance of accurately distinguishing between these "ghosts" and genuinely pathological structures. Such differentiation holds immense importance in preventing unnecessary medical interventions.

## 1 Introduction

Transvenous lead extraction (TLE) is a surgical procedure commonly employed for treating infections or non-infectious conditions (such as lead malfunction, device upgrades, and thrombosis) in cardiac implantable electronic devices (CIEDs). This procedure boasts a high success rate and carries a minimal risk of complications [1,2]. According to a study conducted by Diemberger I, out of over 5000 cases of lead extraction, the procedure-related mortality rate was merely 0.4 %, contributing to 11 % of the total mortality. As a result, TLE is widely recognized as a well-established surgical technique [2]. Nonetheless, the existence of residual tissue following the procedure, referred to as "ghosts", has aroused concerns among clinicians. These "ghosts" typically manifest as fibrous remnants visible after TLE [3]. Ghosts are commonly categorized into two types: stable ghosts, which firmly adhere to the cardiovascular wall and remain in place, and flying ghosts, which comprise fibrous tissue detached from cardiac structures and move freely within the intracardiac chambers [4]. The existence of these "ghosts" has captured clinical attention due to concerns about their clinical relevance and possible complications. While previous case reports and limited research have documented the occurrence of ghosts [5,6], the exact triggering factors and their implications for prognosis remain not entirely understood. This study presents a case of successful identification of a ghost in the right atrium (RA), and explores the occurrence and examination of ghosts, potential factors influencing their emergence, and strategies to mitigate the lingering presence of ghosts following TLE.

### 1.1 Case

**Fig. 1:**
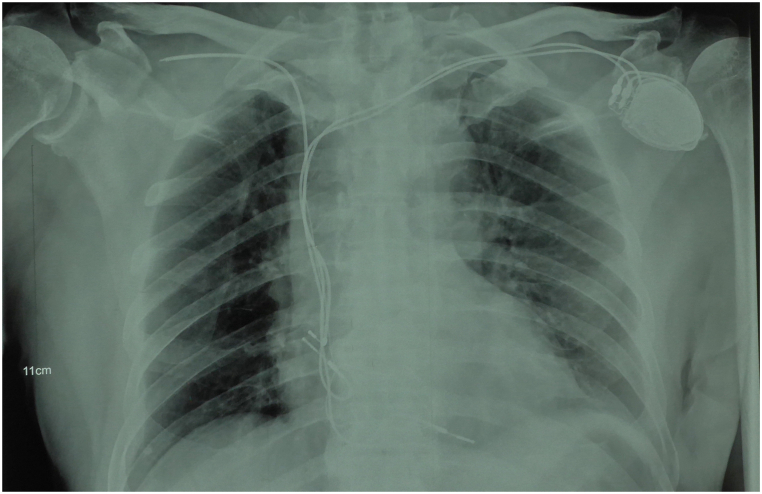
The Chest X-ray demonstrates the course of the residual pacemaker leads.

**Fig. 2:**
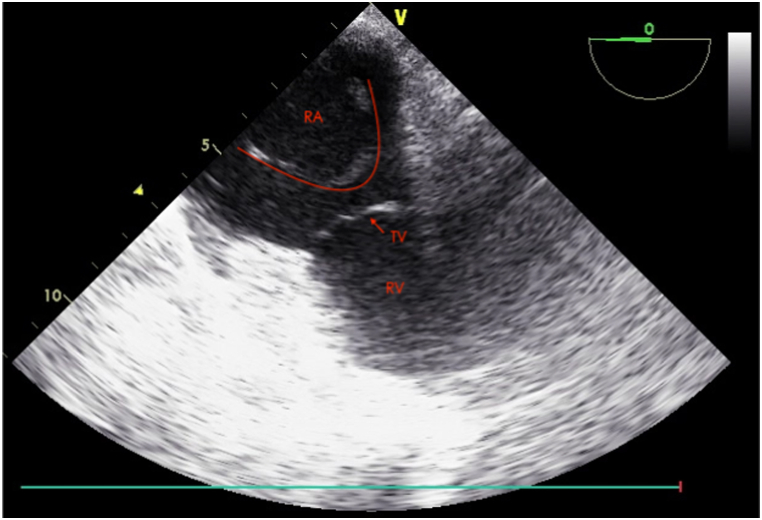
Ghost in the right atrium (red curve); RA: right atrium; TV: tricuspid valve; RV: right ventricle.

**Fig. 3:**
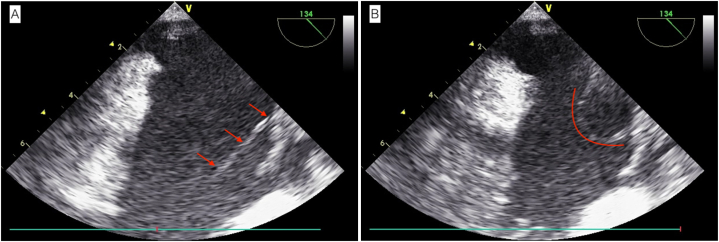
Modified bi-caval view; 3A (left) and 3B (right) illustrate the morphology of the ghost.

A 75-year-old male was admitted to the hospital due to two months of persistent fever. This elderly man underwent permanent dual-chamber pacemaker (DDD type) implantation surgery 10 years ago due to sick sinus syndrome. One year ago, he experienced an infection in his pacemaker pocket and subsequently underwent open chest pacemaker removal surgery while retaining the leads. Two months ago, his surgical wound became ulcerated with purulent discharge accompanied by chills and fever. He was diagnosed with a Staphylococcus aureus infection at another hospital and received antimicrobial treatment. The initial diagnosis included sick sinus syndrome, pacemaker pocket infection following permanent pacemaker implantation, and infective endocarditis, with no other complications present in the patient. Upon admission, the patient had no fever, and his white blood cell count was 8.57 × 10^9/L with a C-reactive protein level of 41.20 mg/L. Blood cultures showed no abnormalities. The chest X-ray did not reveal any pulmonary abnormalities, but it demonstrated the course of the pacemaker leads as shown in Fig. 1. The electrocardiogram revealed a pacing rhythm, and transthoracic echocardiography (TTE) indicated the presence of pacemaker-related vegetations, along with reduced left ventricular diastolic function and an ejection fraction of 69.3 %. After six days of antimicrobial therapy, the patient underwent general anesthesia for TLE, during which four leads were removed—two atrial leads and two ventricular leads. However, following the extraction of the leads, a TEE examination revealed a long, strip-shaped vegetation in the right atrium, previously referred to as the 'ghost' (Fig. 2, Video 1). Subsequently, we adjusted the views of the TEE to comprehensively examine this ghost from different perspectives. In the modified bi-caval view, we observed that one end of the ghost was attached to the atrial wall, while the other end was freely floating within the blood flow, and the entire ghost exhibited a strip-shaped and curled appearance, oscillating back and forth (see Fig. 3 and Video 2). Under TEE guidance, the ghost underwent comprehensive evaluation and was deemed to have no significant impact, thus no further intervention was deemed necessary. The entire procedure went smoothly. Postoperatively, the patient's white blood cell count was 10.73 × 10^9^/L, and blood cultures remained negative. After 10 days of antimicrobial therapy, the patient was discharged. The patient provided informed consent for the publication of his anonymized case details and images.

## 2 Discussion

This study reports a case of the successful identification of a ghost in the right atrium following pacemaker lead extraction. Through the assistance of intraoperative TTE and TEE, we were able to identify the pacemaker vegetation preoperatively and comprehensively examine the entire ghost within the right atrium immediately. Nevertheless, doctors frequently encounter ghosts incidentally, presenting them with treatment challenges. We will thoroughly explore this topic in the subsequent discussion.

According to the 2018 EHRA expert consensus statement, the success of lead extraction surgery is categorized into two levels: complete procedural success and clinical procedural success. Complete procedural success refers to the successful removal of all target leads. Conversely, clinical procedural success pertains to cases where only a minor portion of residual material is retained during surgery, having no detrimental impact on the outcome [1]. In clinical practice, certain technical challenges, such as vascular reactions around the leads, thrombus formation along the lead pathway, or lead material characteristics, may diminish the likelihood of achieving complete procedural success [7]. Therefore, clinical procedural success is often considered an alternative goal in a hybrid operating room. The term "ghosts" prior to lead extraction refers to fibrous connective tissue surrounding the leads. These fibrous tissues amplify the complexity of lead extraction and reduce the chances of achieving complete procedural success. Given the intricate nature post cardiac device implantation, the guidelines suggest that TEE should be routinely conducted to examine device-related structures during the perioperative period [7,8]. Consequently, as indicated in the research, the majority of identified "ghosts" were detected through TEE examinations. While some variability exists in the research, it is apparent that the occurrence of "ghosts" appears to be relatively frequent. A prospective study unveiled that roughly 14 % of patients exhibited residual intracardiac masses [9]. Another study involving 580 patients undergoing lead extraction found that ghosts were discovered in 19 % of the patients [10]. Even in a study encompassing 1103 patients, an astonishing 44.67 % were found to have ghosts [4].

While the risk factors for the development of ghosts remain unclear, current research suggests that younger patient age at the time of initial device implantation, instances of local infections and infective endocarditis, the proliferation of scar tissue or lead abrasion around the leads, and the number of leads are linked to the occurrence of ghosts [10]. Prior investigations have proposed that ghosts might have the potential to dislodge and obstruct the main pulmonary artery or its branches, ultimately resulting in hemodynamic collapse. Nevertheless, a growing body of research indicates that the implications of ghosts might not be as alarming as previously presumed [11]. In a study by Neto V et al., a ghost was observed in a patient 19 years post pacemaker implantation after lead removal. Conservative treatment was administered, and the patient remained asymptomatic during follow-up [5]. Similarly, El-Zein RS et al. detected a pre-existing "ghost" in a 72-year-old male's right atrium before subcutaneous implantable cardioverter defibrillator placement, with no specific treatment. Three months later, the "ghost" disappeared on echocardiogram, and at one-year follow-up, no recurrence was observed [6]. In a recent prospective study involving 936 patients who underwent preoperative TEE examinations, various forms of excessive connective tissue growth linked to cardiac implantable devices were investigated [12]. These forms included fibrous tissue adhesion between the leads and cardiac structures, lead-to-lead adhesions, and scar tissue surrounding the leads. Remarkably, these phenomena did not independently lead to a decrease in long-term survival [1]. The stable ghost appears to carry a relatively low risk. Conversely, the flying ghost is more prone to fragmenting, which may subsequently be absorbed or cause pulmonary embolism in the smaller pulmonary arteries. However, this usually entails minimal clinical impact. Nonetheless, the presence of any residual material could elevate the risk of infection and other complications. In such circumstances, physicians must assess and judiciously determine further management based on the patient's specific condition and the nature of the residue [13].

Incorporating both the aforementioned cases and the case detailed in this article, a conservative course of action is pursued in managing instances of ghosts. In essence, the current consensus indicates that the presence of "ghosts" does not impact prognosis. The crucial concern lies in the misidentification of the "ghost" as a pathological structure, which could lead to unnecessary interventions. Therefore, as suggested by Poterala M et al., it is recommended that patients post temporal lobe epilepsy surgery undergo echocardiographic examinations to detect any potential remnants of fibrotic tissue [10].

Though there is currently no specific treatment for ghosts, physicians have demonstrated a dedicated commitment to enhancing lead extraction procedures and related management. This comprehensive approach encompasses adept management of the operator, instruments, and collaborative teamwork [14]. The operating physician must undergo specialized training in cardiac electrophysiology and acquire pertinent qualifications and certifications for conducting lead extraction procedures. To sustain proficiency, a minimum of 15 procedures per year is generally considered the requisite training standard for lead extraction. Challenges in lead extraction frequently manifest with longer implanted leads, particularly those entrenched in the superior vena cava or subclavian veins. In such instances, a hybrid approach is recommended. When warranted, a multi-disciplinary team can participate in the procedure, with an electrophysiologist disengaging the lead from the proximal adhesive site until the right atrium level is reached. Subsequently, the surgical team completes the process. Visualization tools such as TEE, fluoroscopy, or three-dimensional computed tomography (3DCT) can be employed during the surgical procedure to offer an all-encompassing perspective of the leads and adjacent tissues [15]. Starck CT and colleagues introduced an innovative transcatheter suction method for addressing vegetations on CIEDs. This suction device, akin to the veno-venous configuration of extracorporeal membrane oxygenation (ECMO), circumvents open surgery and minimizes complications, thereby potentially affording long-term survival benefits [16]. Currently, conclusive evidence is lacking regarding the impact of anesthesia methods on the outcome and prognosis of lead extraction surgery. Present research does not distinctly endorse any specific type of anesthesia (general anesthesia vs. local anesthesia with or without sedation). However, a specialized team's presence is essential to provide holistic support and respond swiftly to any emergent situations during the procedure [1].

## 3 Conclusion

Residual ghosts can be misdiagnosed as pathological structures, resulting in unnecessary treatment interventions. By leveraging visualization devices, surgeons can attain a thorough comprehension of the ghosts' context and exercise prudent decision-making.

## Ethics approval and consent to participate

Not applicable.

## Consent for publication

Written informed consent was obtained from the patient for publication of this case report and any accompanying images.

## Data availability statement

Data will be made available on request.

### Funding

This work was supported by the Bethune Charitable Foundation (BCF-RF-WSQZTZJ-202011-020).

## CRediT authorship contribution statement

**Yaru Li:** Conceptualization, Data curation, Writing – original draft, Writing – review & editing. **Luyang Jiang:** Conceptualization, Data curation. **Lu Wang:** Conceptualization, Investigation. **Qiaoyu Han:** Data curation, Methodology. **Xinrui Yin:** Data curation, Investigation. **Yi Feng:** Conceptualization, Supervision, Visualization.

## Declaration of competing interest

The authors declare that they have no competing interests.

## Footnotes

Appendix A Supplementary data to this article can be found online at https://doi.org/10.1016/j.heliyon.2023.e21982.

## References

[1] Bongiorni M.G., Burri H., Deharo J.C., Starck C., Kennergren C., Saghy L., Rao A., Tascini C., Lever N., Kutarski A., Fernandez Lozano I., Strathmore N., Costa R., Epstein L., Love C., Blomstrom-Lundqvist C., Group E.S.C.S.D. (2018). EHRA expert consensus statement on lead extraction: recommendations on definitions, endpoints, research trial design, and data collection requirements for clinical scientific studies and registries: endorsed by APHRS/HRS/LAHRS. Europace. 2018.

[2] Diemberger I., Biffi M., Lorenzetti S., Martignani C., Raffaelli E., Ziacchi M., Rapezzi C., Pacini D., Boriani G. (2018). Predictors of long-term survival free from relapses after extraction of infected CIED. Europace.

[3] Andreas M., Wiedemann D., Kocher A., Khazen C. (2013). Materialization of ghosts: severe intracardiac masses after pacemaker lead extraction requiring immediate surgical intervention. Heart Rhythm.

[4] Nowosielecka D., Jachec W., Polewczyk A., Tulecki L., Stefanczyk P., Kutarski A. (2022). Ghost", a Well-Known but Not Fully Explained Echocardiographic Finding during Transvenous Lead Extraction: Clinical Significance. Int J Environ Res Public Health.

[5] Neto V., Santos J., Craveiro N., Santos L., Correia M. (2023). A 'ghost' after transvenous intracardiac lead extraction. Neth. Heart J..

[6] El-Zein R.S., Stelzer M., Hatanelas J., Goodlive T.W., Amin A.K. (2020). A ghost left behind after transvenous lead extraction: a finding to be feared. Am J Case Rep.

[7] Nowosielecka D., Jachec W., Polewczyk A., Tulecki L., Kleinrok A., Kutarski A. (2021). The role of transesophageal echocardiography in predicting technical problems and complications of transvenous lead extractions procedures. Clin. Cardiol..

[8] Nowosielecka D., Jachec W., Polewczyk A., Kleinrok A., Tulecki L., Kutarski A. (2021). The prognostic value of transesophageal echocardiography after transvenous lead extraction: landscape after battle. Cardiovasc. Diagn. Ther..

[9] Narducci M.L., Di Monaco A., Pelargonio G., Leoncini E., Boccia S., Mollo R., Perna F., Bencardino G., Pennestri F., Scoppettuolo G., Rebuzzi A.G., Santangeli P., Di Biase L., Natale A., Crea F. (2017). Presence of 'ghosts' and mortality after transvenous lead extraction. Europace.

[10] Poterala M., Kutarski A., Brzozowski W., Tomaszewski M., Gromadzinski L., Tomaszewski A. (2020). Echocardiographic assessment of residuals after transvenous intracardiac lead extraction. Int. J. Cardiovasc. Imag..

[11] Caiati C., Luzzi G., Pollice P., Favale S., Lepera M.E. (2020). A novel clinical perspective on new masses after lead extraction (ghosts) by means of intracardiac echocardiography. J. Clin. Med..

[12] Nowosielecka D., Jachec W., Polewczyk A., Tulecki L., Kleinrok A., Kutarski A. (2021). Prognostic value of preoperative echocardiographic findings in patients undergoing transvenous lead extraction. Int. J. Environ. Res. Publ. Health.

[13] Wazni O., Wilkoff B.L. (2016). Considerations for cardiac device lead extraction. Nat. Rev. Cardiol..

[14] Kusumoto F.M., Schoenfeld M.H., Wilkoff B.L., Berul C.I., Birgersdotter-Green U.M., Carrillo R., Cha Y.M., Clancy J., Deharo J.C., Ellenbogen K.A., Exner D., Hussein A.A., Kennergren C., Krahn A., Lee R., Love C.J., Madden R.A., Mazzetti H.A., Moore J.C., Parsonnet J., Patton K.K., Rozner M.A., Selzman K.A., Shoda M., Srivathsan K., Strathmore N.F., Swerdlow C.D., Tompkins C., Wazni O. (2017). HRS expert consensus statement on cardiovascular implantable electronic device lead management and extraction. Heart Rhythm. 2017.

[15] Kiuchi K., Fukuzawa K., Mori S., Nishii T., Matsumoto K., Ichibori H., Yamada T. (2017). The details of an unusual "ghost" after transvenous lead extraction: three-dimensional computed tomography analysis. J Arrhythm.

[16] Starck C.T., Schaerf R.H.M., Breitenstein A., Najibi S., Conrad J., Berendt J., Esmailian F., Eulert-Grehn J., Dreizler T., Falk V. (2020). Transcatheter aspiration of large pacemaker and implantable cardioverter-defibrillator lead vegetations facilitating safe transvenous lead extraction. Europace.

